# Discrete and Polymeric, Mono- and Dinuclear Silver Complexes of a Macrocyclic Tetraoxime Ligand with Ag^I^–Ag^I^ Interactions

**DOI:** 10.3390/s130505671

**Published:** 2013-05-02

**Authors:** Shohei Tashiro, Jun-ichiro Tanihira, Mihoko Yamada, Mitsuhiko Shionoya

**Affiliations:** Department of Chemistry, Graduate School of Science, the University of Tokyo, 7-3-1 Hongo, Bunkyo-ku, Tokyo 113-0033, Japan; E-Mails: tashiro@chem.s.u-tokyo.ac.jp (S.T.); tanihira@chem.s.u-tokyo.ac.jp (J.T.); myamada@chem.eng.osaka-u.ac.jp (M.Y.)

**Keywords:** silver, macrocycle, oxime, crystal, metal-metal interaction

## Abstract

Macrocyclic compounds that can bind cationic species efficiently and selectively with their cyclic cavities have great potential as excellent chemosensors for metal ions. Recently, we have developed a tetraoxime-type tetraazamacrocyclic ligand **1** formed through a facile one-pot cyclization reaction. Aiming to explore and bring out the potential of the tetraoxime macrocycle **1** as a chelating sensor, we report herein the preparation of several kinds of silver complexes of **1** and their unique coordination structures determined by single-crystal X-ray diffraction analyses. As a result, the formation of two kinds of discrete structures, monomeric complexes [Ag(**1**)X] (X = counter anions) and a dimeric complex [Ag_2_(**1**)_2_]X_2_, and two kinds of polymeric structures from a mononuclear complex, [Ag(**1**)]*_n_*X*_n_*, and from a dinuclear complex, [Ag_2_(**1**)X_2_]*_n_*, was demonstrated. In the resulting complexes, the structurally flexible macrocyclic ligand **1** was found to provide several different coordination modes. Notably, in some silver complexes of **1**, Ag^I^–Ag^I^ interactions were observed with different Ag^I^–Ag^I^ distances which depend on the kind of counter anions and the chemical composition.

## 1. Introduction

In the molecular design of chemosensors, macrocyclic compounds provide excellent structural and functional motifs due to their efficient and specific binding to analytes through multipoint interactions within restricted cyclic skeletons. So far, a great number of macrocyclic compounds such as crown ethers [1], cryptands [2], cyclic polyamines [3], porphyrins [4] and Schiff-base macrocycles [5] have been extensively used for cation [6] and anion [7] sensing by covalently attached chromophores, luminophores [8–10] or redox-active moieties [11], and for indicator-displacement assays (IDAs) in which an indicator molecule non-covalently bound to a host molecule is displaced by an analyte to cause signaling [12]. Recently, arrangement or immobilization of macrocycles on several interfaces or nanometer-sized materials allows for more practical sensing applications [13–15]. Therefore, while selection and combination of the signaling moieties are obviously important, it will also be necessary to develop novel chemically modifiable macrocyclic skeletons capable of binding analytes in well-defined modes.

Previously we reported the synthesis of a tetraoxime-type tetraazamacrocycle, 3,7,11,15-tetra-methyl-1,5,9,13,2,6,10,14-tetraoxatetraazacyclohexa-2,6,10,14-tetraene (**1**), by facile metal-template one-pot cyclization of a simple dioxime-type compound [16]. The macrocyclic ligand **1** can complex with various divalent transition metal ions such as Fe^II^, Ni^II^, Cu^II^ and Pd^II^ to form six-coordinate octahedral and four-coordinate square-planar structures. Notably, macrocycle **1** has a flexible, larger 16-membered cyclic skeleton compared with widely-used tetraazamacrocycles such as a 12-membered cyclen and a 14-membered cyclam. Moreover, the oxime nitrogen donors of **1** generally show lower Lewis basicity than nitrogen donors such as amine, imine and hydrazone. A unique coordination structure based on these features was preliminarily found in a crystalline, linear polymeric Ag^I^ complex in which Ag_2_L_1_-type dinuclear complexes are bridged by counter anions [16].

**Figure 1.**
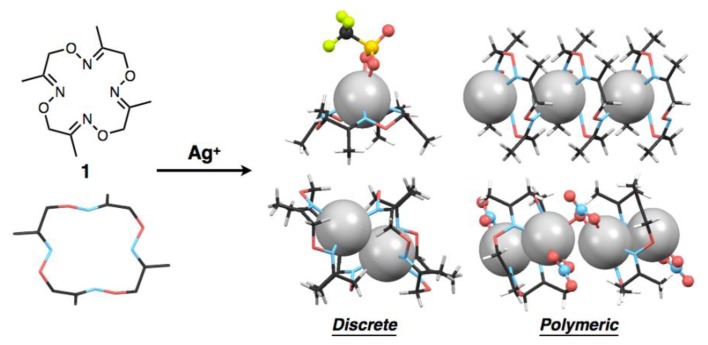
Schematic representation of the formation of four types of Ag^I^ complexes of **1** with discrete or polymeric structures.

As part of the development of the metal binding ability of **1** as a basic structure of chemosensors, we found that ligand **1**, depending on the kind of Ag^I^ salts, forms a variety of discrete or polymeric Ag^I^ complexes with different compositional ratios such as [Ag(**1**)], [Ag_2_(**1**)_2_] and [Ag_2_(**1**)] in the crystalline state (Figure 1). We also found Ag^I^–Ag^I^ interactions in some crystal structures, in which the distances between two Ag^I^ ions vary with the kind of counter anions and/or the ligand-to-metal ratios. It is well known that metal-metal interactions of d^10^ metals such as Cu^I^, Ag^I^ and Au^I^ ions and square planar d^8^ metals such as Rh^I^, Pd^II^ and Pt^II^ ions produce characteristic luminescence with appropriate ligands [17–21]. Although we have not so far observed such behaviour of the Ag^I^ complexes of **1**, ligands capable of mediating metal-metal interactions would have great potential to serve as a luminescent chemosensor with appropriate bridging ligands or additional pendant ligand on the macrocycle.

## 2. Experimental Section

### 2.1. General Procedures

All solvents, organic and inorganic reagents except for the macrocyclic ligand **1** are commercially available, and were used without further purification. The ligand **1** was synthesised and isolated as colorless crystals via formation of the ferrous complex, [Fe(**1**)(CH_3_CN)_2_] (ClO_4_)_2_, according to our previous report [16]. Crystallisation of silver complexes was carried out in the dark. Single-crystal X-ray crystallographic analyses were performed using a Rigaku RAXIS-RAPID imaging plate diffractometer with MoK*α* (*λ* = 0.71075 Å) radiation, and data obtained were calculated using the CrystalStructure crystallographic software package except for refinement, which was performed using SHELXL-97 [22]. Crystallographic data and refinement details for obtained five complexes are shown in Table 1. The X-ray structures are displayed using the Mercury program.

### 2.2. Crystallisation of [Ag(**1**)NO_3_]

Ligand **1** (1.0 mg, 3.5 μmol) and AgNO_3_ (5.9 mg, 35 μmol) were dissolved in CH_3_CN (2 mL). The resulting solution was slowly evaporated for 2 days at room temperature. Colorless block crystals appeared with a powder of excess silver salts. One of the crystals was analysed by single-crystal X-ray diffraction measurement.

### 2.3. Crystallisation of [Ag(**1**)CF_3_SO_3_]

Ligand **1** (1.0 mg, 3.5 μmol) and AgCF_3_SO_3_ (9.0 mg, 35 μmol) were dissolved in CH_3_CN (1 mL). After addition of toluene (1 mL), the resulting mixed solution was slowly evaporated for 1 week at room temperature. Colorless plate crystals appeared with powder of excess silver salts. One of the crystals was analysed by single-crystal X-ray diffraction measurement.

### 2.4. Crystallisation of [Ag_2_(**1**)_2_] (SbF_6_)_2_

Ligand **1** (0.10 mg, 0.35 μmol) and AgSbF_6_ (0.60 mg, 1.75 μmol) were dissolved in CHCl_3_ (0.25 mL). The mixed solution was crystallised by slow diffusion of diethyl ether as a poor solvent at room temperature. After several days, colorless block crystals appeared and one of the crystals was analysed by single-crystal X-ray diffraction measurement.

### 2.5. Crystallisation of [Ag(**1**)]_n_(BF_4_)_n_

Ligand **1** (0.23 mg, 0.81 μmol) and AgBF_4_ (1.6 mg, 8.2 μmol) were dissolved in CH_3_CN (0.25 mL). After addition of toluene (0.25 mL), the resulting mixed solution was slowly evaporated for several days at room temperature. Colorless block crystals appeared with a powder of excess silver salts. One of the crystals was analysed by single-crystal X-ray diffraction measurement.

### 2.6. Crystallisation of [Ag_2_(**1**)(NO_3_)_2_]_n_

Ligand **1** (1.0 mg, 3.5 μmol) and AgNO_3_ (5.9 mg, 35 μmol) were dissolved in CH_3_CN (1 mL). After addition of toluene (1 mL), the resulting mixed solution was slowly evaporated for 1 week at room temperature. Colorless plate crystals appeared with a powder of excess silver salts. One of the crystals was analysed by single-crystal X-ray diffraction measurement.

## 3. Results

### 3.1. Two Discrete, Mononuclear Silver Complexes, [Ag(**1**)NO_3_] and [Ag(**1**)CF_3_SO_3_]

**Figure 2.**
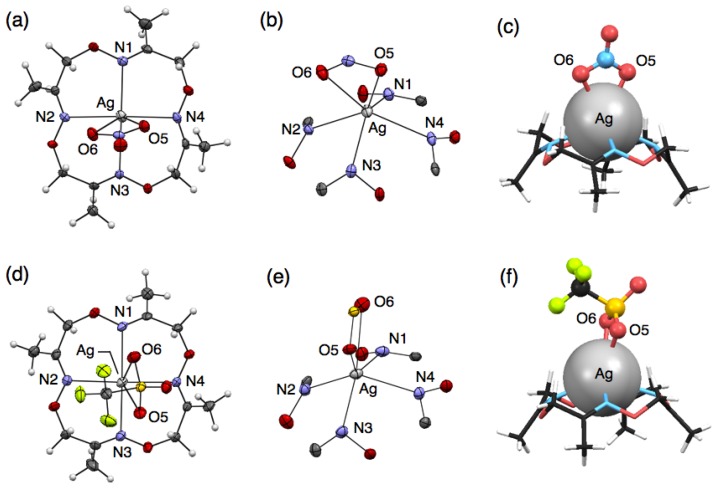
Crystal structures of (**a**–**c**) [Ag(**1**)NO_3_] and (**d**–**f**) [Ag(**1**)CF_3_SO_3_]. (**a**,**d**) Top views and (**b**,**e**) coordination structures represented with 50% thermal ellipsoids; (**c**,**f**) Side views represented with space fill (Ag), stick (**1**) and ball-stick (counter anions) models.

To precisely evaluate the molecular structures of silver complexes of **1** by single-crystal X-ray diffraction analysis, we examined crystallisation of mixed solutions of **1** with several kinds of silver salts. Crystallisation of the mixed solution of **1** with 10 equivalents of AgNO_3_ or AgCF_3_SO_3_ salts afforded similar mononuclear complexes, [Ag(**1**)NO_3_] or [Ag(**1**)CF_3_SO_3_], respectively. For example, when ligand **1** and AgNO_3_ were dissolved in CH_3_CN, colorless block crystals were obtained after slow evaporation. Single-crystal X-ray analysis of the resulting block crystal revealed the molecular structure of a neutral, mononuclear silver complex, [Ag(**1**)NO_3_] (Figure 2(a)). In this structure, four nitrogen atoms of **1** and two oxygen atoms of an NO_3_^−^ ion bind to an Ag^I^ ion to form a six-coordinate structure (Figure 2(b)). The bound Ag^I^ ion does not fit into the N_4_-plane of **1**, but sits on the four upturned nitrogen atoms of **1**, resulting in the formation of a shuttlecock-like structure (Figure 2(c)). The structure of [Ag(**1**)NO_3_] is chiral so that both enantiomers exist in the crystal as a racemate.

Crystallisation of a mixed solution of **1** with AgCF_3_SO_3_ in CH_3_CN-toluene produced a similar complex, [Ag(**1**)CF_3_SO_3_] (Figure 2(d)). This structure also has a shuttlecock-like structure with a six-coordinate Ag^I^ ion bound by four nitrogen atoms of **1** and two oxygen atoms of a CF_3_SO_3_^−^ anion (Figure 2(e,f)). This chiral complex [Ag(**1**)CF_3_SO_3_] also exists as a racemate in the crystal. In comparing Ag–N bond distances in both complexes, [Ag(**1**)NO_3_] and [Ag(**1**)CF_3_SO_3_], an average Ag–N distance of 2.490 Å for [Ag(**1**)CF_3_SO_3_] is significantly shorter than that of 2.536 Å for [Ag(**1**)NO_3_] (Tables 2 and 3). In contrast, an average Ag–O distance of 2.641 Å for [Ag(**1**)CF_3_SO_3_] is significantly longer than that of 2.513 Å for [Ag(**1**)NO_3_]. This is probably due to the difference in the basicities of both anions, CF_3_SO_3_^−^ and NO_3_^−^.

### 3.2. A Discrete, Dimeric Silver Complex, [Ag_2_(**1**)_2_] (SbF_6_)_2_

**Figure 3.**
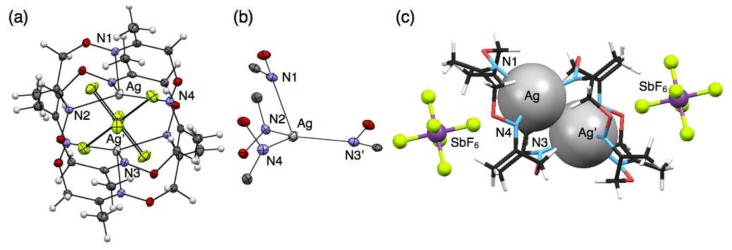
Crystal structure of [Ag_2_(**1**)_2_] (SbF_6_)_2_. (**a**) A top view; and (**b**) the coordination structure represented with 50% thermal ellipsoids; (**c**) A side view represented with space fill (Ag), stick (**1**) and ball-stick (counter anions) models.

Crystallisation of a solution of **1** and five equivalents of AgSbF_6_ in CHCl_3_ yielded colorless block crystals composed of a cationic, dimeric silver complex, [Ag_2_(**1**)_2_] (SbF_6_)_2_, as proven by single-crystal X-ray analysis (Figure 3(a)). In this structure, an Ag^I^ ion sits on the three upturned nitrogen atoms of **1** to form a shuttlecock-like structure which is similar to the structures of [Ag(**1**)NO_3_] and [Ag(**1**)CF_3_SO_3_]. However, another one nitrogen atom N(3) of **1** binds to another Ag^I^ ion to form a dimeric complex [Ag_2_(**1**)_2_]^2+^ with a four-coordinate Ag^I^ ion (Figure 3(b)). The average Ag–N distance of 2.421 Å for [Ag_2_(**1**)_2_]^2+^ (Table 4) is obviously shorter than those of six-coordinate [Ag(**1**)NO_3_] and [Ag(**1**)CF_3_SO_3_]. Notably, the resulting dimeric structure shows Ag^I^–Ag^I^ interactions with a distance of 3.364 Å, which is shorter than the sum of van der Waals radii (Ag–Ag = 3.44 Å) [23,24]. The dimeric structure with a pair of Ag^I^ ions is composed of an enantiomeric pair arising from the chiral conformation of **1**. In this crystal, the non-coordinating anions SbF_6_^−^ do not bind to Ag^I^, but are accommodated in the bowl-shaped pocket of **1** surrounded by four methyl groups (Figure 3(c)).

### 3.3. A Polymeric Silver Complex Based on Mononuclear Units, [Ag(**1**)]_n_(BF_4_)_n_

**Figure 4.**
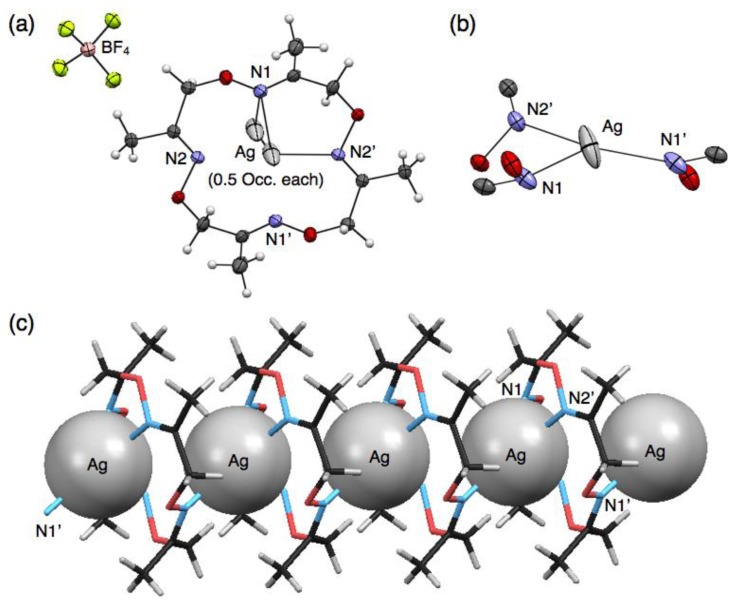
Crystal structure of [Ag(**1**)]*_n_*(BF_4_)*_n_*. (**a**) A top view; and (**b**) the coordination structure represented with 50% thermal ellipsoids; (**c**) A side view represented with space fill (Ag) and stick (**1**) models. BF_4_^−^ anions and disordered Ag^I^ cations are omitted for clarity in (**b**) and (**c**).

In contrast to the discrete structures described above, a polymeric, cationic silver complex, [Ag(**1**)]*_n_*(BF_4_)*_n_*, was obtained from complexation of **1** with 10 equivalents of AgBF_4_. Upon slow evaporation of a mixed solution of both components in CH_3_CN-toluene, colorless block crystals were obtained suitable for single-crystal X-ray analysis. In the crystal structure, ligand **1** adopts a non-chiral stair-like conformation and binds one Ag^I^ ion that is disordered between two positions that are 1.466 Å apart (0.5 occupancy each) (Figure 4(a)). The Ag^I^ ion adopts a three-coordinate geometry with two shorter and one longer Ag–N distances of 2.249, 2.359 and 2.630 Å, respectively (Table 5). For three nitrogen atoms bound to one Ag^I^ ion, two nitrogen atoms come from one ligand and the third nitrogen atom from another neighboring ligand (Figure 4(b)), resulting in the connection of two macrocycles through one Ag^I^ ion to form a polymeric structure (Figure 4(c)). Due to the disorder of Ag^I^, although three kinds of Ag^I^–Ag^I^ distances are possible (3.190, 4.396 and 5.726 Å), the second shortest distance of 4.396 Å appears to be more probable. The one-dimensional Ag^I^ arrays are arranged parallel in the crystal packing structure. Counter anions, BF_4_^–^, also are arranged linearly in the interstitial channels which are formed by the parallel packing of Ag^I^ arrays.

### 3.4. A Polymeric Silver Complex Based on Dinuclear Units, [Ag_2_(**1**)(NO_3_)_2_]_n_

**Figure 5.**
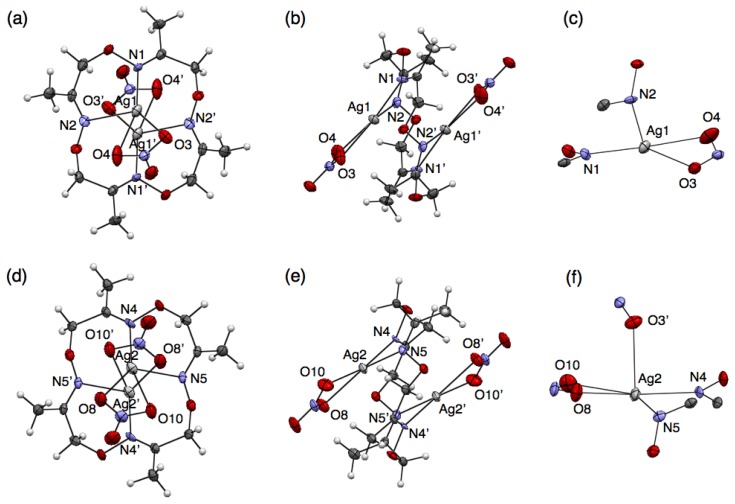
Crystal structure of [Ag_2_(**1**)(NO_3_)_2_]*_n_*. (**a**–**c**) One [Ag_2_(**1**)(NO_3_)_2_] structure containing Ag(1); and (**d**–**f**) another [Ag_2_(**1**)(NO_3_)_2_] structure containing Ag(2). (**a**,**d**) Top views, (**b**,**e**) side views and (**c**,**f**) coordination structures represented with 50% thermal ellipsoids.

Another polymeric silver complex was found as plate crystals from a mixed solution of **1** and 10 equivalents of AgNO_3_ in CH_3_CN-toluene, which are different conditions from those used with the mononuclear complex, [Ag(**1**)NO_3_], formed from the CH_3_CN solution as block crystals. The single-crystal X-ray analysis of the plate crystal revealed a unit structure of a neutral, dinuclear silver complex, [Ag_2_(**1**)(NO_3_)_2_], for a one-dimensional coordination polymer, [Ag_2_(**1**)(NO_3_)_2_]*_n_*. The stair-like conformation of **1** is almost the same as [Ag(**1**)]*_n_*(BF_4_)*_n_*, thus **1** in [Ag_2_(**1**)(NO_3_)_2_]*_n_* can also bind two Ag^I^ ions to form a polymeric structure. Notably, the coordination polymer is composed of two kinds of [Ag_2_(**1**)(NO_3_)_2_] units. In one [Ag_2_(**1**)(NO_3_)_2_], the Ag(1) atom adopts a four-coordinate structure with two nitrogen atoms of the same **1** and two oxygen atoms of one NO_3_^−^ anion (Figure 5(a–c)). Meanwhile, the other [Ag_2_(**1**)(NO_3_)_2_] contains a five-coordinate Ag(2) atom bound by two nitrogen atoms of **1**, two oxygen atoms of one NO_3_^−^ anion and one oxygen atom of another NO_3_^−^ (Figure 5(d–f)). Importantly, both [Ag_2_(**1**)(NO_3_)_2_] structures show Ag^I^–Ag^I^ interactions with different distances (Ag(1)–Ag(1′): 3.235 Å, Ag(2)–Ag(2′): 3.348 Å) (Table 6).

**Figure 6.**
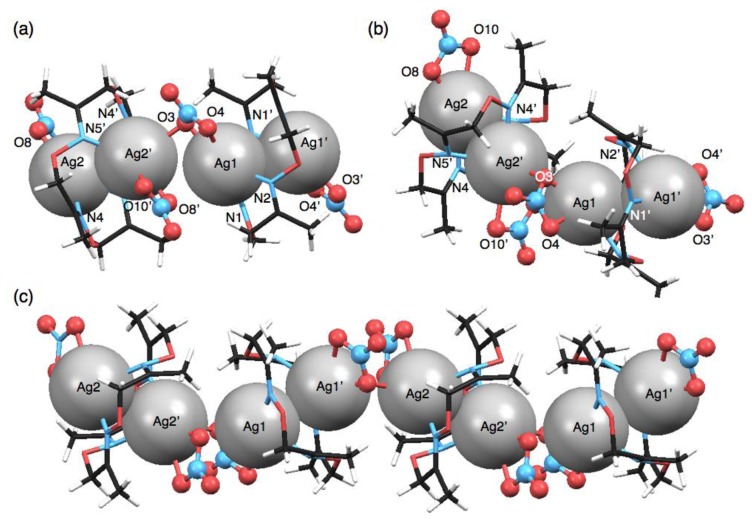
Polymeric structure of [Ag_2_(**1**)(NO_3_)_2_]*_n_* in the crystal. (**a**) A top view; and (**b**) a side view of a crookedly connected structure containing two [Ag_2_(**1**)(NO_3_)_2_. [units; and (**c**) a polymeric structure represented with space fill (Ag), stick (**1**) and ball-stick (counter anions) models.

Both [Ag_2_(**1**)(NO_3_)_2_] units are crookedly connected with each other through an O(3) atom of one NO_3_^−^ anion (Figure 6(a,b)). The inter-unit Ag^I^–Ag^I^ distance of 3.709 Å is relatively short. As the result of alternate arrangement of two [Ag_2_(**1**)(NO_3_)_2_] units, Ag^I^ ions are aligned in a zigzag fashion (Figure 6(c)). In the crystal packing structure, the coordination polymers are arranged parallel and closely.

## 4. Discussion

### 4.1. Ligand Conformation

**Figure 7.**
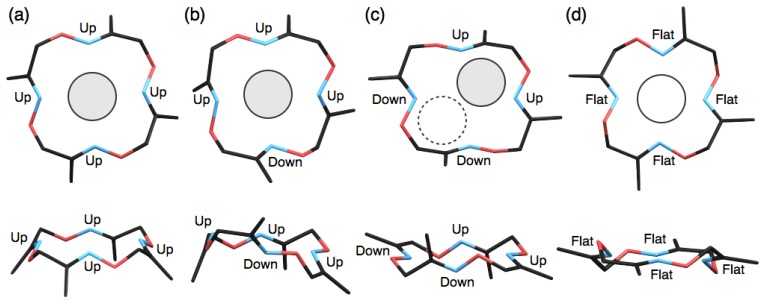
Top and side views of four conformations of ligand **1** in each complex. (**a**) A shuttlecock-like conformation in [Ag(**1**)CF_3_SO_3_]; (**b**) A flipped shuttlecock-like conformation in [Ag(**1**)CF_3_CO_2_] [16]; (**c**) A stair-like conformation in [Ag(**1**)]*_n_*(BF_4_)*_n_*; (**d**) A planar conformation in [Ni(**1**)(CH_3_CN)_2_] (ClO_4_)_2_ [16].

Macrocyclic ligands and their metal complexes such as crown ethers [25] and macrocyclic polyamines [26–28] have been well studied and discussed with regard to their conformation as one of the most important factors to determine their metal complexation behaviours. In this study, we have demonstrated the formation of several types of silver complexes of **1** with different coordination geometries, compositional ratios and packing structures. The main origin for the diverse structures appears to be the relatively flexible macrocyclic structure of **1** capable of adopting several different ring conformations. So far, at least four conformations of **1** were found in the single-crystal X-ray analyses. For example, two mononuclear complexes, [Ag(**1**)NO_3_] and [Ag(**1**)CF_3_SO_3_], and one dimeric complex, [Ag_2_(**1**)_2_] (SbF_6_)_2_, adopt shuttlecock-like conformations (Figure 7(a)). In these cases, Ag^I^ ions sit on the four nitrogen atoms of **1** due to the large ionic radius of Ag^I^. Interestingly, a mononuclear complex, [Ag(**1**)CF_3_CO_2_], previously reported [16] has a similar but significantly different conformation of **1**, that is regarded as a flipped shuttlecock-like conformation in which one nitrogen atom is oriented to the opposite face (Figure 7(b)). The differences in the crystal packing, counter anions and crystallisation conditions might cause the partial flipping of the shuttlecock-like conformation of **1**.

In contrast to the discrete complexes, the present polymeric complexes, [Ag(**1**)]*_n_*(BF_4_)*_n_* and [Ag_2_(**1**)(NO_3_)_2_]*_n_*, and previously reported [Ag_2_(**1**)(CF_3_CO_2_)_2_]*_n_* [16], possess a stair-like conformation of **1**, in which two nitrogen atoms and the other two atoms of **1** are oriented to upper and lower faces, respectively (Figure 7(c)). As the result, one macrocycle **1** can bind two Ag^I^ ions on both faces simultaneously to form polymeric structures. It should be also noted that the stair-like conformation is quite similar to the conformation of metal-free ligand **1** [16], suggesting that the polymer formation of silver complexes of **1** seems to be less strained, predictable structures.

The last planar conformation of **1** found previously [16] is similar to those of conventional tetraaza macrocyclic complexes (Figure 7(d)). In our previous study, this conformation was exclusively observed with divalent transition metal complexes in a six-coordinate octahedral geometry such as [Fe(**1**)(CH_3_CN)_2_] (ClO_4_)_2_, [Ni(**1**)(CH_3_CN)_2_] (ClO_4_)_2_, [Ni(**1**)(H_2_O)_2_] (ClO_4_)_2_, [Cu(**1**)(ClO_4_)_2_] and [Pd(**1**)] (BF_4_)_2_ [16]. This result indicates that the combination of larger Ag^I^ ions and the structurally flexible ligand **1** allows the formation of diverse and unique silver complexes. This finding is expected to provide further information on further functionalisation of tetraoxime macrocycle **1** as a metal-selective chemosensor or a template for multi-metal assemblies.

### 4.2. Ag^I^–Ag^I^ Interactions and Ag^I^ Arrays

**Figure 8.**
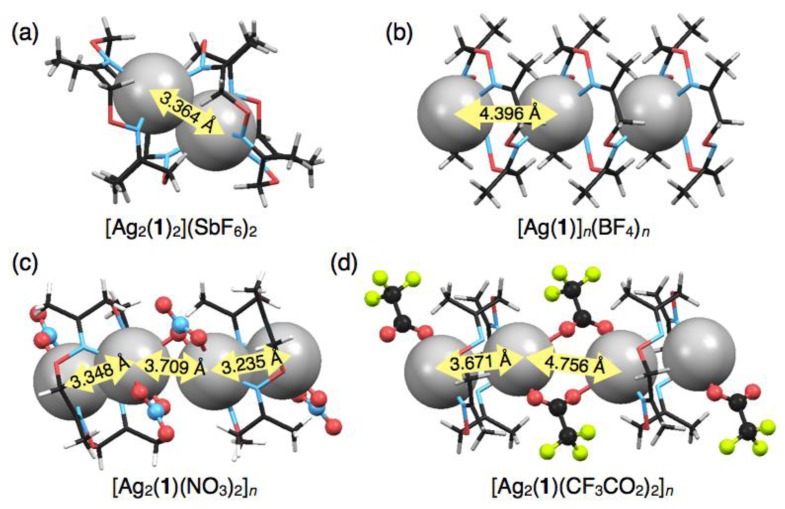
Several Ag^I^ arrangement structures in (**a**) [Ag_2_(**1**)_2_] (SbF_6_)_2_; (**b**) [Ag(**1**)]*_n_*(BF_4_)*_n_*; (**c**) [Ag_2_(**1**)(NO_3_)_2_]*_n_*; and (**d**) [Ag_2_(**1**)(CF_3_CO_2_)_2_]*_n_* [16].

Construction of Ag^I^–Ag^I^ bonded Ag^I^ chain arrays in crystals has attracted much attention in the fields of crystal engineering, supramolecular chemistry and metal–organic frameworks, because the Ag^I^ ion possesses flexible coordination geometries and labile and diverse bonding manners such as Ag^I^–N, Ag^I^–π, Ag^I^–Ag^I^ [29,30]. Therefore, a novel molecular platform for Ag^I^-based coordination polymers would make a significant contribution to this research area. In this study, several types of crystalline Ag^I^ complexes were constructed, in which the Ag^I^–Ag^I^ distances varied due to the structural flexibility of **1**. For example, in three mononuclear discrete complexes, [Ag(**1**)NO_3_], [Ag(**1**)CF_3_SO_3_] and [Ag(**1**)CF_3_CO_2_], Ag^I^ ions are chemically isolated. On the other hand, the dimeric complex, [Ag_2_(**1**)_2_] (SbF_6_)_2_, possesses an isolated Ag^I^-pair with Ag^I^–Ag^I^ interaction (Ag^I^–Ag^I^ distance: 3.364 Å) (Figure 8(a)). The polymeric complex based on mononuclear units, [Ag(**1**)]*_n_*(BF_4_)*_n_*, does not include any Ag^I^–Ag^I^ interactions, but forms a one-dimensional Ag^I^ array in the crystal [Figure 8(b)]. In contrast, the polymeric complex based on dinuclear units, [Ag_2_(**1**)(NO_3_)_2_]*_n_*, allows the formation of an Ag^I^ array involving two different Ag^I^–Ag^I^ distances of 3.235 and 3.348 Å (Figure 8(c)). Interestingly, a polymeric complex, [Ag_2_(**1**)(CF_3_CO_2_)_2_]*_n_*, with different counter anions forms a similar Ag^I^ array but with longer Ag^I^–Ag^I^ distances of 3.671 and 4.756 Å (Figure 8(d)) [16]. These results suggest that the alignment properties of Ag^I^ complexes in the crystal states would vary with the coordinating counter anions of silver salts. In addition, crystallisation condition is an important factor to determine the crystal packing structures of the silver complexes of **1**.

### 4.3. Prospects for Chemosensors Using **1**

Finally, prospects for chemosensors using **1** are the development of different types of crystals of mononuclear, dinuclear and polymeric silver complexes with various Ag^I^–Ag^I^ distances depending on the kind of counter anions. If each crystal exhibits optical and electrical properties specific to the structural factors such as nucleus number and metal-metal distance, ligand **1** would serve as an anion sensor through metal-induced crystallisation. Due to its structural flexibility, ligand **1** can also complex with various transition metal ions such as Fe^II^, Ni^II^, Cu^II^, Pd^II^ and Ag^I^ [16], suggesting the potential of **1** as cation sensors or other functional materials as demonstrated in recent progress of coordination chemistry [31,32].

## 5. Conclusions

In this study, we demonstrated the structural diversity of tetraoxime macrocyclic silver complexes to bring out the potential of **1** as a macrocyclic chemosensor. Single-crystal X-ray analyses revealed the molecular structures of five silver complexes of **1**. Depending on the kinds of counter anions and ring conformation of **1**, two discrete mononuclear complexes, [Ag(**1**)NO_3_] and [Ag(**1**)CF_3_SO_3_], one discrete dimeric complex, [Ag_2_(**1**)_2_] (SbF_6_)_2_, one polymeric complex based on mononuclear units, [Ag(**1**)]*_n_*(BF_4_)*_n_* and one polymeric complex based on dinuclear units, [Ag_2_(**1**)(NO_3_)_2_]*_n_*, were constructed. The crystal packing structures of these complexes possessed different arrangement patterns of Ag^I^ ions. Importantly, some silver complexes can induce Ag^I^–Ag^I^ interactions in the crystals. These results unambiguously demonstrate the structural diversity of **1** leading to a new macrocyclic platform for chemosensors.

## Figures and Tables

**Table 1.** Crystallographic data and refinement details for [Ag(**1**)NO_3_], [Ag(**1**)CF_3_SO_3_], [Ag_2_(**1**)_2_](SbF_6_)_2_, [Ag(**1**)]*_n_*(BF_4_)*_n_* and [Ag_2_(**1**)(NO_3_)_2_]*_n_*.

	**[Ag(1)NO_3_]**	**[Ag(1)CF_3_SO_3_]**	**[Ag_2_(1)_2_] (SbF_6_)_2_**	**[Ag(1)]***_n_***(BF_4_)***_n_*	**[Ag_2_(1)(NO_3_)_2_]***_n_*
Formula	C_12_H_20_AgN_5_O_7_	C_13_H_20_AgF_3_N_4_O_7_S	C_12_H_20_AgF_6_N_4_O_4_Sb	C_12_H_20_AgBF_4_N_4_O_4_	C_12_H_20_Ag_2_N_6_O_10_
Formula weight	454.19	541.25	627.92	478.99	624.06
Crystal system	triclinic	triclinic	orthorhombic	tetragonal	triclinic
Space group	*P*–1	*P*–1	*Pbca*	*P*4/*n*	*P*–1
*a* (Å)	8.4448(14)	8.442(2)	16.0247(4)	19.4458(15)	8.0660(11)
*b* (Å)	10.5207(16)	9.988(3)	13.4358(3)	19.4458(15)	9.6775(13)
*c* (Å)	11.576(2)	12.974(3)	17.7726(5)	4.3965(4)	13.3698(15)
*α* (°)	67.188(5)	71.262(8)	90	90	67.686(4)
*β* (°)	81.490(4)	88.062(7)	90	90	85.264(4)
*γ* (°)	63.784(3)	69.975(7)	90	90	84.658(5)
*V* (Å^3^)	850.2(3)	969.8(4)	3826.51(17)	1662.5(3)	960.0(2)
*Z*	2	2	8	4	2
*T* (K)	93	93	93	108	93
*D*_calcd_ (g/cm^3^)	1.774	1.853	2.180	1.914	2.159
*μ* (mm^−1^)	1.228	1.217	2.514	1.279	2.101
2*θ*_max_ (°)	55	55	55	55	55
Reflns, total	8344	9464	34764	22040	9440
Reflns, unique	3841	4400	4384	1904	4341
*R*_int_	0.0267	0.0372	0.0313	0.0600	0.0505
*R*_1_ (*I* > 2σ(*I*)) a	0.0241	0.0332	0.0179	0.1003	0.0560
*wR*_2_ (all data) b	0.0626	0.1017	0.0403	0.2600	0.1743
GOF on *F*^2^	1.083	1.131	1.180	1.118	1.118
CCDC numbers	918055	918056	918057	918058	918059

a *R*_1_ = ∑‖*F_o_*| – |*F_c_*‖/∑|*F_o_*|;

b *wR*_2_ = [∑ [*w*(*F_o_*^2^ – *F_c_*^2^)^2^]/∑*w*(*F_o_*^2^)^2^]]^1/2^.

**Table 2.** Selected bond lengths (Å) and angles (deg) for [Ag(**1**)NO_3_].

Ag(1)–N(1)	2.471(2)	Ag(1)–N(2)	2.530(3)
Ag(1)–N(3)	2.5945(17)	Ag(1)–N(4)	2.5476(15)
Ag(1)–O(5)	2.430(3)	Ag(1)–O(6)	2.5967(17)
N(1)–Ag(1)–N(2)	83.96(7)	N(1)–Ag(1)–N(3)	136.31(7)
N(1)–Ag(1)–N(4)	81.03(6)	N(1)–Ag(1)–O(5)	118.69(7)
N(1)–Ag(1)–O(6)	125.67(6)	N(2)–Ag(1)–N(3)	79.73(7)
N(2)–Ag(1)–N(4)	133.00(7)	N(2)–Ag(1)–O(5)	134.35(5)
N(2)–Ag(1)–O(6)	83.44(7)	N(3)–Ag(1)–N(4)	81.17(5)
N(3)–Ag(1)–O(5)	101.31(7)	N(3)–Ag(1)–O(6)	92.41(5)
N(4)–Ag(1)–O(5)	91.41(7)	N(4)–Ag(1)–O(6)	139.97(8)
O(5)–Ag(1)–O(6)	50.94(6)		

**Table 3.** Selected bond lengths (Å) and angles (deg) for [Ag(**1**)CF_3_SO_3_].

Ag(1)–N(1)	2.489(3)	Ag(1)–N(2)	2.460(4)
Ag(1)–N(3)	2.512(3)	Ag(1)–N(4)	2.498(3)
Ag(1)–O(5)	2.588(3)	Ag(1)–O(6)	2.693(3)
N(1)–Ag(1)–N(2)	83.44(10)	N(1)–Ag(1)–N(3)	139.05(8)
N(1)–Ag(1)–N(4)	83.41(10)	N(1)–Ag(1)–O(5)	135.80(9)
N(1)–Ag(1)–O(6)	83.08(8)	N(2)–Ag(1)–N(3)	83.09(11)
N(2)–Ag(1)–N(4)	139.73(8)	N(2)–Ag(1)–O(5)	123.30(9)
N(2)–Ag(1)–O(6)	116.62(9)	N(3)–Ag(1)–N(4)	82.41(10)
N(3)–Ag(1)–O(5)	82.90(8)	N(3)–Ag(1)–O(6)	137.13(9)
N(4)–Ag(1)–O(5)	91.77(9)	N(4)–Ag(1)–O(6)	99.27(9)
O(5)–Ag(1)–O(6)	54.28(8)		

**Table 4.** Selected bond lengths (Å) and angles (deg) for [Ag_2_(**1**)_2_] (SbF_6_)_2_.

Ag(1)–N(1)	2.4203(17)	Ag(1)–N(2)	2.4999(16)
Ag(1)–N(3′)	2.3320(17)	Ag(1)–N(4)	2.4307(18)
Ag(1)–Ag(1′)	3.3636(3)		
N(1)–Ag(1)–N(2)	83.56(6)	N(1)–Ag(1)–N(3′)	115.83(6)
N(1)–Ag(1)–N(4)	84.69(6)	N(2)–Ag(1)–N(3′)	108.38(6)
N(2)–Ag(1)–N(4)	126.29(6)	N(3′)–Ag(1)–N(4)	123.82(6)

**Table 5.** Selected bond lengths (Å) and angles (deg) for [Ag(**1**)]*_n_*(BF_4_)*_n_*.

Ag(1)–N(1)	2.249(5)	Ag(1)–N(1′)	2.359(6)
Ag(1)–N(2′)	2.630(5)		
N(1)–Ag(1)–N(1′)	143.0(3)	N(1)–Ag(1)–N(2′)	85.13(17)
N(1′)–Ag(1)–N(2′)	122.60(16)		

**Table 6.** Selected bond lengths (Å) and angles (deg) for [Ag_2_(**1**)(NO_3_)_2_]*_n_*.

Ag(1)–N(1)	2.318(8)	Ag(1)–N(2)	2.493(6)
Ag(1)–O(3)	2.334(7)	Ag(1)–O(4)	2.647(8)
Ag(2)–N(4)	2.373(7)	Ag(2)–N(5)	2.429(6)
Ag(2)–O(3′)	2.630(7)	Ag(2)–O(8)	2.338(6)
Ag(2)–O(10)	2.705(7)	Ag(1)–Ag(1′)	3.2351(9)
Ag(2)–Ag(2′)	3.3481(8)	Ag(1)–Ag(2′)	3.7090(8)
N(1)–Ag(1)–N(2)	81.7(3)	N(1)–Ag(1)–O(3)	143.3(2)
N(1)–Ag(1)–O(4)	163.1(3)	N(2)–Ag(1)–O(3)	134.9(3)
N(2)–Ag(1)–O(4)	85.4(3)	O(3)–Ag(1)–O(4)	50.6(2)
N(4)–Ag(2)–N(5)	80.1(2)	N(4)–Ag(2)–O(3′)	88.0(2)
N(4)–Ag(2)–O(8)	143.9(2)	N(4)–Ag(2)–O(10)	162.7(3)
N(5)–Ag(2)–O(3′)	115.0(3)	N(5)–Ag(2)–O(8)	134.6(3)
N(5)–Ag(2)–O(10)	90.4(2)	O(3′)–Ag(2)–O(8)	84.8(3)
O(3′)–Ag(2)–O(10)	83.0(3)	O(8)–Ag(2)–O(10)	49.9(3)
